# Intake of *Lactobacillus paragasseri* SBT2055 improves subjective symptoms of common cold during winter season in healthy adults: A randomized, double-blind, placebo-controlled parallel-group comparative study

**DOI:** 10.3389/fnut.2022.1063584

**Published:** 2022-12-08

**Authors:** Eiji Kobatake, Yoshitaka Iwama, Toshinobu Arai, Nobuhiko Shioya, Mai Kise, Toshihide Kabuki

**Affiliations:** ^1^Milk Science Research Institute, MEGMILK SNOW BRAND Co., Ltd., Saitama, Japan; ^2^Nihonbashi Cardiology Clinic, Tokyo, Japan; ^3^Research and Development Planning Department, MEGMILK SNOW BRAND Co., Ltd., Tokyo, Japan; ^4^KSO Corporation, Tokyo, Japan; ^5^Products Development Department, MEGMILK SNOW BRAND Co., Ltd., Saitama, Japan

**Keywords:** *Lactobacillus paragasseri* SBT2055, probiotics, clinical study, subjective symptoms, common cold, salivary sIgA, oxidative stress

## Abstract

**Objective:**

*Lactobacillus paragasseri* SBT2055 (LG2055) has been reported to show immunostimulating effects. This study aimed to investigate the effects of LG2055 on the subjective symptoms of the physical condition in healthy adults.

**Materials and methods:**

In this randomized, double-blind, placebo-controlled, parallel-group comparative study, Japanese individuals aged 20–64 years were recruited. A total of 200 participants were randomly divided into two groups by an independent controller (LG2055 and placebo groups; 100 participants per group). Drinkable yogurts containing LG2055 or lacking LG2055 (placebo) were used as test samples. The participants ingested one bottle of the test sample once a day for 12 weeks. A daily physical health questionnaire survey (about common cold symptoms) was performed as the primary outcome, and immunological and oxidative stress markers in saliva and serum were evaluated as secondary outcomes.

**Results:**

In total, 198 participants completed the scheduled intake of the test samples, and five participants were excluded from the final analysis. Consequently, 193 participants (LG2055 group, *n* = 97; placebo group, *n* = 96) in the Per-Protocol Set were included in the efficacy analysis. The cumulative days of each symptom were evaluated, and the LG2055 group showed a significantly higher ratio of “without symptom” in runny nose, plugged nose, sneezing, sore throat, hoarseness, cough, headache, feeling tired, and fever than the placebo group, indicating that the incidence rates of common cold symptoms were lower in the LG2055 group. Additionally, changes in the salivary secretory IgA levels were significantly higher, and the serum derivatives of reactive oxygen metabolites levels were significantly lower in the LG2055 group.

**Conclusion:**

Our study revealed that intake of LG2055 decreased common cold symptoms and improved immune parameters in healthy adults. This suggests that LG2055 contributes to the maintenance of physical conditions by improving the host immune system.

**Clinical trial registration:**

[https://www.umin.ac.jp/ctr/index.htm], identifier [UMIN000045901].

## Introduction

Common cold is the most familiar disease across all generations worldwide, and its symptoms include local symptoms occurring mainly in the upper respiratory tract (e.g., cough, runny nose, plugged nose, sore throat, and sneezing) and systemic symptoms (e.g., fever, chill, and feeling tired). Common cold is caused by infection of microorganisms, mainly viruses, in the upper respiratory tract (1). As many viruses can cause common cold, immediate identification of the causative virus is difficult. Thus, symptomatic treatment and adequate rest are mainly advised for curing common cold. In addition, common cold induces several social problems, such as increased medical expenditure, decreased productivity, and economic loss due to absence from work.

Notably, maintenance of the biological defense system could be effective in preventing common cold. Considering the spread of severe acute respiratory syndrome coronavirus 2 (SARS-CoV-2) infection presently, it is important that the immune systems be maintained and improved in everyday life (2). It is presumed that food ingredients are useful for the continuous improvement of the biological defense system because we can easily ingest them daily. In fact, in recent years, the immunostimulating effects of various food ingredients, such as lactic acid bacteria (LAB) (3), Bifidobacterium (4), lactoferrin (5), and polyphenols (6, 7), have been demonstrated.

Of note, many studies have investigated the availability and functionality of various strains of LAB in recent years, and the most common function of LAB is improvement of the intestinal environment; other functions including, immunomodulatory, anti-obesity, and anti-inflammatory effects, have also been reported. Interestingly, accumulating evidence indicates that the beneficial effects of LAB on the host are achieved by various mechanisms. Probiotics, which are defined as “live microorganisms that, when administrated in adequate amounts, confer health benefits on the host (8),” are the most commonly known functional LAB. In recent years, it has been reported that not only probiotics, but also non-viable bacteria, bacterial components, extracts, or metabolites obtained from bacteria could provide health benefits to the host (9). Thus, the concept of postbiotics, which are defined as the “preparation of inanimate microorganisms and/or their components that confers a health benefit on the host,” has been proposed (10).

*Lactobacillus paragasseri* SBT2055 (LG2055) originates from the human intestine and is a type of probiotic LAB. A recent study has revealed that *Lactobacillus gasseri* strains are divided into two groups, and some *Lactobacillus gasseri* strains, including LG2055, are reclassified as *Lactobacillus paragasseri* sp. nov. (11). Notably, LG2055 has a high tolerance to bile acid (12) and the ability to become established in the intestine (13, 14), and the safety of LG2055 ingestion has been confirmed by clinical studies (15, 16). In addition, it has been used for manufacturing dairy products in Japan, and is hence regarded as a safe food ingredient. Many studies have been conducted on LG2055, and its beneficial effects, such as improvement of the intestinal environment (17), prevention of abdominal adiposity (18–20), and reduction of oxidative stress (21, 22), have been demonstrated.

Moreover, it has been reported that LG2055 has immunostimulating effects. Our previous studies demonstrated the beneficial effects of LG2055 *in vivo*, such as induction of immunoglobulin A (IgA) production (23) and prevention of viral infection (24, 25). Furthermore, a clinical study showed that the intake of drinkable yogurt (DY) containing LG2055 increased hemagglutination inhibition titers against influenza virus after vaccination, total IgG and IgA levels in plasma, secretory IgA (sIgA) production in saliva, natural killer (NK) cell activity, and anti-viral gene expression (16). These findings suggest that LG2055 activates both innate and adaptive human immune responses and contributes to strengthening the biological defense system of the host.

People with a weakened immune system, caused by stress, poor sleep, or undernutrition, are more likely to get sick, and weakening of the immune system is associated not only with diseases, such as cancer and infectious diseases, but also with the quality of life, including fatigue and sleep disturbances (26, 27). Thus, it is essential to maintain and improve the immune system.

Accordingly, this randomized, double-blind, placebo-controlled, parallel-group comparative study aimed to evaluate whether the intake of DY containing LG2055 is effective in the maintenance and improvement of immune systems and whether LG2055 could maintain a good physical condition in healthy adults.

## Materials and methods

### Study design

**FIGURE 1:**
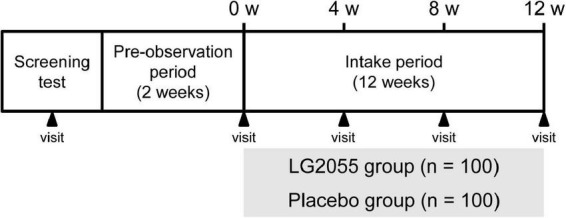
Schematic representation of the study protocol.

This randomized, double-blind, placebo-controlled, parallel-group comparative study was conducted from October 2021 to April 2022. The study protocol was approved by the Institutional Review Board of the Ethics Committee of Nihonbashi Cardiology Clinic (Tokyo, Japan) (approval date: 27 September 2021; approval number: NJI-021-09-01). This study was registered in the University Hospital Medical Information Network Clinical Trials Registry prior to its initiation (UMIN ID: UMIN000045901) and conducted according to the principles of the Helsinki Declaration of 1975 as revised in 2013 and the Ethical Guidelines for Medical and Health Research Involving Human Subjects proposed by the Ministry of Education, Culture, Sports, Science and Technology and the Ministry of Health, Labour and Welfare of Japan. Participants provided written informed consent before the initiation of the study. This study included a screening test, a pre-observation period of 2 weeks, and an intake period of 12 weeks (Figure 1). Participants answered the physical health questionnaire daily and were interviewed by the medical doctor five times (during the screening test and 0, 4, 8, and 12 weeks after starting intake) during this study.

### Participants

In this study, Japanese males and females (aged 20–64 years) who tended to catch a cold or get poor physical conditions were recruited. In the recruitment process, we asked the candidates a question about the frequency of catching a cold. Those who more often catch a cold were considered to be appropriate for this study and selected as the participants. On the other hand, those who seldom catch a cold were considered to be unsuitable for the participants.

Participants with one or more of the following criteria were excluded from participation: (1) participants suffering from, undergoing treatment for, or with a history of serious diseases, such as diabetes, kidney/liver disease, heart disease or thyroid disease, adrenal disease, and other metabolic diseases; (2) participants with chronic diseases and who took medication on a daily basis; (3) participants who had been diagnosed with dry mouth; (4) participants who were unable to abstain from taking supplement, food for specified health use or functional food, or health food that may affect immune function; (5) participants who were unable to abstain from taking food containing LAB, Bifidobacterium, oligosaccharides, or viable bacteria during the study period; (6) participants who consistently drank more than an appropriate amount of alcohol; (7) participants who were unable to abstain from alcohol for 2 days prior to the screening test and each test; (8) participants with food allergies; (9) participants who took or planned to take medicine for seasonal allergic rhinitis (pollen allergy); (10) participants with digestive diseases affecting digestion and absorption and those with a history of digestive surgery (excluding appendicitis); (11) participants who tended to get diarrhea by taking dairy products; (12) pregnant women, women who intended to become pregnant during the research period, and women who were breastfeeding; (13) participants who were judged to be inappropriate as research participants based on blood tests results obtained during the screening tests; (14) participants who had a history or current condition of drug or alcohol dependence; (15) participants who were participating in research involving the ingestion of other foods or the use of other medicines or those who had participated in or were willing to participate in other clinical research within 1 month of obtaining consent; (16) participants who were judged to be inappropriate as research participants by the principal investigator; (17) participants who smoked 21 or more cigarettes a day; (18) participants who planned to receive the influenza vaccine 3 weeks before ingestion to the end of the ingestion period; (19) participants who planned to receive the coronavirus disease 2019 (COVID-19) vaccine during the ingestion period; (20) participants who worked night shifts; (21) participants who planned to travel abroad, including overseas travel, during the study; and (22) participants who had donated more than 200 ml of blood within 1 month or 400 ml of blood within 3 months prior to the date of obtaining consent, or those who had donated blood components.

Throughout the study, the participants were instructed for the following observances: (1) intake the test samples as instructed; (2) do not allow other persons to intake the test samples; (3) avoid drinking alcohol 2 days before the test; (4) avoid taking any food and drinks after 21:00 on the day before the test (only water was permitted); (5) avoid taking water 1 h before the test and until the end of the test; (6) avoid smoking until the end of the test on the day of the test; (7) avoid any dental treatment 2 days before the test; (8) maintain your regular lifestyle, such as food and exercise (to avoid undereating, overeating, overexercising, and traveling abroad); (9) avoid taking more food or drinks, including caffeine, than usual; (10) avoid the use and/or the intake of medicines, supplements, and/or healthy food (including Food for Specified Health Uses and Foods with Functional Claims) that may influence the immune system; (11) avoid the intake of food containing viable bacteria, such as LAB, Bifidobacteria, and natto (fermented soybeans) bacteria, and/or enhanced with oligosaccharides; (12) avoid donation of blood and/or blood components; (13) avoid the overconsumption of alcohol (up to 20 g alcohol/day); (14) use medicines after getting the permission of the principal investigator (except in case of emergency); and (15) keep a daily record of the test sample consumption, defecation, ingestion of healthy food, usage of medicine, and physical health questionnaire.

In addition, during the study period, SARS-CoV-2 infection had spread rapidly worldwide, and vaccination against SARS-CoV-2 was being promoted by the government in Japan. Therefore, we moralistically permitted the participants to get inoculated; however, we instructed them to avoid getting inoculated 1 week before the blood sampling.

### Test samples

We prepared two types of DYs as test samples—an active DY containing LG2055 and a placebo DY lacking LG2055, as described in our previous study (16). The active DY was prepared with yogurt starter cultures (*Streptococcus thermophilus*) commonly used for conventional yogurt production and viable cells of LG2055. A DY mixture consisting of approximately 10% dairy product and a small amount of flavoring agent, a stabilizer, and an artificial sweetener, was inoculated with the yogurt starter cultures and LG2055 cells and then fermented. The active DY contained a minimum of 1 × 10^9^ cfu/100 g of LG2055; whereas, although the placebo DY was prepared in the same manner, the LG2055 cells were not added. Each DY was identical in energy (36 kcal), protein (3.3 g), fat (0 g), carbohydrate (5.5 g), and sodium (43 mg) content per 100 g; all the DYs had the same flavor and were indistinguishable by taste. The test DYs were kept in cold storage and delivered weekly.

The participants ingested one bottle of active or placebo DY once a day for 12 weeks.

### Outcomes

In this study, a daily physical health questionnaire survey (about local and systemic symptoms associated with the common cold) was performed as the primary outcome. In addition, salivary sIgA (concentration, secretion rate, and amount of secretion), NK cell activity, serum IgA level, serum IgG level, serum derivatives of reactive oxygen metabolites (d-ROMs) level, and serum biological antioxidant potential (BAP) level were evaluated as secondary outcomes. For safety assessment, body weight (body mass index), blood pressure, pulse, hematological test, biochemical test, urinalysis, doctor’s consultation, and adverse events were surveyed.

#### Physical health questionnaire

The daily physical health questionnaire was designed according to the Wisconsin Upper Respiratory Symptom Survey (WURSS)-21, WURSS-24 (28), and previous reports (29, 30). During the intake period, the participants recorded their subjective symptoms (local symptoms, including runny nose, plugged nose, sneezing, sore throat, hoarseness, cough, and headache, and systemic symptoms, including feeling tired, chill, and fever) in the physical health questionnaire daily. The severity of each symptom was evaluated in five grades (No symptom, Very mild, Mild, Moderate, and Severe) (Supplementary Figure 1). The survey was conducted in the Japanese language.

#### Salivary secretory IgA

Saliva samples were collected during the screening tests and at 0, 4, 8, and 12 weeks after the start of intake. Saliva was collected using cotton swabs and Salivette devices for 2 min. The amount of saliva secreted per minute was calculated. Saliva samples were frozen and stored until analysis. The concentration of sIgA in saliva was measured using Human s-IgA (Saliva) ELISA Kit (Yanaihara Institute Inc., Shizuoka, Japan) according to the manufacturer’s instructions.

#### Natural killer cell activity

Blood samples were collected at 0, 4, 8, and 12 weeks after starting intake. NK cell activity was measured by a chromium-51 (^51^Cr) release assay at an effector to target ratio of 50:1. For the effector cells, peripheral blood mononuclear cells were separated from blood samples by density gradient centrifugation. For the target cells, K562 cells were labeled by ^51^Cr (PerkinElmer Japan, Yokohama, Japan). The target cells were added to the effector cells, and the cells were cultured at 37°C in 5% CO_2_ for 4 h. The released ^51^Cr was measured by Wizard2 Automatic Gamma Counter (PerkinElmer, MA, USA). NK cell activity was calculated from the results and expressed as percentage cytotoxicity.

#### Serum immunoglobulin A and immunoglobulin G

Blood samples were collected in the screening tests and at 0, 4, 8, and 12 weeks after starting intake. The concentrations of serum IgA and IgG were measured by turbidimetric immunoassay using the N-assay TIA IgA-SH, and N-assay TIA IgG-SH (Nitobo, Tokyo, Japan) according to the manufacturer’s instructions.

#### Serum derivatives of reactive oxygen metabolites and biological antioxidant potential

Blood samples were collected at 0 and 12 weeks after starting intake. The concentrations of serum d-ROMs and BAP were measured using FREE CARRIO DUO (Diacron International, Grosscto, Italy) and a reagent kit according to the manufacturer’s instructions.

#### Safety assessment

In the screening tests and at 0, 4, 8, and 12 weeks after starting intake, subjective and objective symptoms were determined by the doctor’s consultation. The following parameters were measured in the participants’ blood and urine samples in the screening tests and at 0 and 12 weeks after starting intake. Hematological parameters included white and red blood cell counts, hemoglobin, hematocrit, and blood platelets. Biochemical parameters included total protein, albumin, aspartate aminotransferase, alanine transaminase, lactate dehydrogenase, total bilirubin, alkaline phosphatase, gamma-glutamyl-transpeptidase, blood urea nitrogen, uric acid, sodium, chloride, potassium, total cholesterol, high-density lipoprotein cholesterol, low-density lipoprotein cholesterol, triglycerides, glucose, and hemoglobin A1c (HbA1c). Urinalysis parameters included glucose (qualitative), protein (qualitative), urobilinogen (qualitative), bilirubin (qualitative), pH, ketones, and occult blood. These measurements were performed by LSI Medience Corporation (Tokyo, Japan). Participants were instructed to keep a daily record of the test sample consumption, defecation, diarrhea, menstruation, outing, usage of medicine, and physical condition during the pre-observation and intake periods.

### Sample size

We determined the sample size of this study using data derived from our previous clinical study (16). Based on the data of the salivary sIgA secretion rate, more than 89 participants in each group were necessary to detect a difference between groups at a 5% significance level and statistical power of 80%. Thus, a total of 100 participants were included in each group in this study.

### Randomization

Participants were divided into two groups by block randomization. Randomization was performed based on sex, age, salivary sIgA, serum IgA, serum IgG, Profile of Mood States 2nd Edition (POMS2), influenza vaccination, and COVID-19 vaccination status. Subsequently, an independent controller assigned each group to the LG2055 or placebo group. The controller sealed the assignment list and kept it sealed until the designated time for unmasking.

### Statistical analysis

Based on the physical health questionnaire, the cumulative days of each symptom during the intake period were assessed by the chi-square test.

For the immunological and oxidative stress markers, the change from baseline (0 week) to each subsequent time point (4, 8, and 12 weeks) was calculated, and the average and the standard deviation were calculated for each marker. Shapiro-Wilk test was used to evaluate the normal distribution of each data. The between-group comparisons were performed by an unpaired *t*-test in the case of normal distribution and by the Wilcoxon rank sum test in the case of non-normal distribution. For within-group comparisons between 0 week and each subsequent time point (4, 8, and 12 weeks), Dunnett’s test was applied in the case of normal distribution, and Wilcoxon signed-rank test with Bonferroni correction was applied in the case of non-normal distribution.

Statistical analyses were performed using IBM SPSS Statistics 27. A *p* value of <0.05 was considered statistically significant.

## Results

### Participants

**FIGURE 2:**
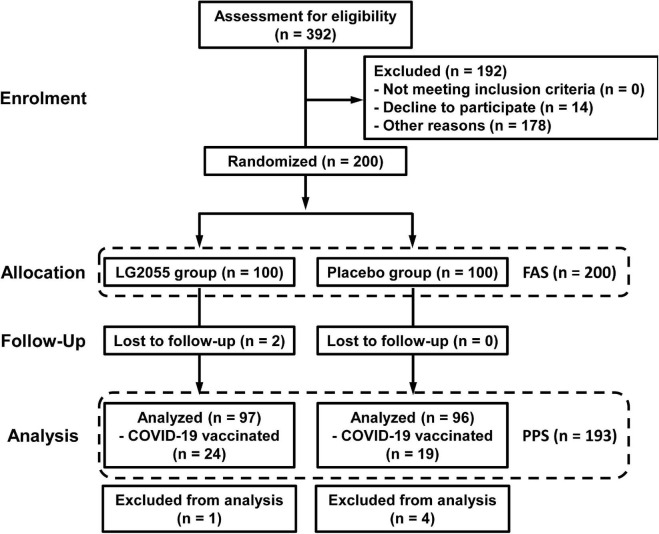
Flow of participants through each stage of this study. For the participants who got COVID-19 vaccination during the pre-observation and intake periods, a part of the data of daily physical health questionnaire survey was excluded.

The flow of participant selection is shown in Figure 2. The recruitment of the participants began in September 2021. A total of 200 participants, who were considered to be appropriate, were selected from 392 candidates, and they were finally enrolled in this study. They were randomized into two groups (LG2055 group: *n* = 100; placebo group: *n* = 100) and began to intake test samples. During the intake period, two participants discontinued the trial because of SARS-CoV-2 infection or decline; thus, 198 participants completed the scheduled intake of the test samples. Subsequently, five participants were excluded because of the intake of medicines that might have influenced the immune system during the study period. As a result, 193 participants (LG2055 group: *n* = 97; placebo group: *n* = 96) in the Per-Protocol Set (PPS) were included in the efficacy analysis. In addition, 43 participants (24 participants in the LG2055 group and 19 participants in the placebo group) got vaccination against SARS-CoV-2 during the pre-observation and intake periods. There was no significant difference in the ratio of participants who got inoculated between the two groups.

### Background of participants

The background of the participants is shown in Table 1. Between the two groups, the HbA1c level was significantly different between the LG2055 and placebo groups (5.4 ± 0.3, vs. 5.3 ± 0.3, *p* = 0.039); however, it was judged not to affect the results by the medical doctor. There was no significant between-group difference for the other markers. In addition, the levels of the hematological and biochemical markers in each group were within the normal range (data not shown).

**TABLE 1:** Background of participants.

		LG2055 group	Placebo group	*p* value
		*n* = 100	*n* = 100	
Sex	Male/Female	43/57	42/58	0.886
Age	Years	43.7 ± 11.7	43.1 ± 11.2	0.735
Height	cm	163.8 ± 9.1	163.5 ± 8.2	0.797
Weight	kg	60.1 ± 11.8	60.3 ± 10.4	0.887
BMI	kg/m^2^	22.3 ± 3.1	22.5 ± 2.6	0.643
				(Mean ± SD)

### Ingestion rate

Among the 198 participants who completed the scheduled intake of the test samples, the ingestion rate was over 97%, and a statistically significant difference between the two groups was not detected.

### Primary outcome

Because some participants got the vaccination against SARS-CoV-2 during the pre-observation and intake periods, several symptoms (headache, feeling tired, chill, and fever) were excluded from the questionnaire results for 7 days following vaccination to eliminate the influence of side effects from the vaccine on the physical health questionnaire.

The cumulative days of symptoms (five grades) are shown in Table 2. Statistically significant differences were observed in all symptoms, indicating that the distributions of the severity of these symptoms were different between the two groups. Therefore, the severity of each symptom was converted into two grades (“No symptom” was converted to “without symptom,” and “Very mild,” “Mild,” “Moderate,” and “Severe” were converted to “with symptom”), and the cumulative days of symptoms were evaluated. The LG2055 group showed a significantly higher ratio of “without symptom” in runny nose, plugged nose, sneezing, sore throat, hoarseness, cough, headache, feeling tired, and fever than the placebo group (Table 3), indicating that the incidence rates of symptoms associated with the common cold were lower in the LG2055 group. Although the incidence rates were different among the symptoms, decreased incidence rates in the LG2055 group were detected for multiple symptoms. Especially, “Very mild” and “Mild” symptoms seemed to have decreased in the LG2055 group. Moreover, the LG2055 group showed a significantly lower ratio of “without symptom” in chill.

**TABLE 2:** Comparison of the cumulative days of each symptom (five grade).

Symptoms	Group	*n*	No symptom	Very mild	Mild	Moderate	Severe	*p* value
				
			Cumulative days (Ratio)					
Runny nose	LG2055	8,148	5,764 (70.7%)	1,826 (22.4%)	425 (5.2%)	110 (1.4%)	23 (0.3%)	<0.001*
	Placebo	8,066	5,297 (65.7%)	2,013 (25.0%)	598 (7.4%)	148 (1.8%)	10 (0.1%)	
Plugged nose	LG2055	8,148	6,702 (82.3%)	1,100 (13.5%)	224 (2.7%)	115 (1.4%)	7 (0.1%)	<0.001*
	Placebo	8,066	5,830 (72.3%)	1,658 (20.6%)	433 (5.4%)	120 (1.5%)	25 (0.3%)	
Sneezing	LG2055	8,148	6,704 (82.3%)	1,046 (12.8%)	268 (3.3%)	118 (1.4%)	12 (0.1%)	<0.001*
	Placebo	8,066	6,438 (79.8%)	1,252 (15.5%)	305 (3.8%)	66 (0.8%)	5 (0.1%)	
Sore throat	LG2055	8,148	7,633 (93.7%)	385 (4.7%)	80 (1.0%)	32 (0.4%)	18 (0.2%)	<0.001*
	Placebo	8,066	7,078 (87.8%)	754 (9.3%)	186 (2.3%)	48 (0.6%)	0 (0.0%)	
Hoarseness	LG2055	8,148	7,859 (96.5%)	236 (2.9%)	33 (0.4%)	9 (0.1%)	11 (0.1%)	<0.001*
	Placebo	8,066	7,402 (91.8%)	480 (6.0%)	140 (1.7%)	41 (0.5%)	3 (0.0%)	
Cough	LG2055	8,148	7,709 (94.6%)	331 (4.1%)	71 (0.9%)	35 (0.4%)	2 (0.0%)	<0.001*
	Placebo	8,066	7,393 (91.7%)	508 (6.3%)	145 (1.8%)	16 (0.2%)	4 (0.0%)	
Headache^※1^	LG2055	7,988	7,204 (90.2%)	477 (6.0%)	177 (2.2%)	102 (1.3%)	28 (0.4%)	<0.001*
	Placebo	7,946	6,890 (86.7%)	721 (9.1%)	249 (3.1%)	70 (0.9%)	16 (0.2%)	
Feeling tired^※1^	LG2055	7,988	7,058 (88.4%)	650 (8.1%)	191 (2.4%)	68 (0.9%)	21 (0.3%)	<0.001*
	Placebo	7,946	6,461 (81.3%)	1,058 (13.3%)	241 (3.0%)	136 (1.7%)	50 (0.6%)	
Chill^※1^	LG2055	7,988	7,598 (95.1%)	286 (3.6%)	79 (1.0%)	21 (0.3%)	4 (0.1%)	0.005*
	Placebo	7946	7,629 (96.0%)	255 (3.2%)	51 (0.6%)	11 (0.1%)	0 (0.0%)	
Fever^※1^	LG2055	7,988	7,846 (98.2%)	102 (1.3%)	25 (0.3%)	11 (0.1%)	4 (0.1%)	<0.001*
	Placebo	7,946	7,694 (96.8%)	210 (2.6%)	28 (0.4%)	11 (0.1%)	3 (0.0%)	

*Significant difference was observed between two groups (*p* < 0.05).

^※1^The data for 7 days after COVID-19 vaccination were excluded from the results.

**TABLE 3:** Comparison of the cumulative days of each symptom (two grade).

Symptoms	Group	*n*	With symptom		Without symptom		*p* value
			Cumulative days	Ratio (%)	Cumulative days	Ratio (%)	
Runny nose	LG2055	8,148	2,384	29.3	5,764	70.7	< 0.001*
	Placebo	8,066	2,769	34.3	5,297	65.7	
Plugged nose	LG2055	8,148	1,446	17.7	6,702	82.3	< 0.001*
	Placebo	8,066	2,236	27.7	5,830	72.3	
Sneezing	LG2055	8,148	1,444	17.7	6,704	82.3	< 0.001*
	Placebo	8,066	1,628	20.2	6,438	79.8	
Sore throat	LG2055	8,148	515	6.3	7,633	93.7	< 0.001*
	Placebo	8,066	988	12.2	7,078	87.8	
Hoarseness	LG2055	8,148	289	3.5	7,859	96.5	< 0.001*
	Placebo	8,066	664	8.2	7,402	91.8	
Cough	LG2055	8,148	439	5.4	7,709	94.6	< 0.001*
	Placebo	8,066	673	8.3	7,393	91.7	
Headache^※1^	LG2055	7,988	784	9.8	7,204	90.2	< 0.001*
	Placebo	7,946	1,056	13.3	6,890	86.7	
Feeling tired^※1^	LG2055	7,988	930	11.6	7,058	88.4	< 0.001*
	Placebo	7,946	1,485	18.7	6,461	81.3	
Chill^※1^	LG2055	7,988	390	4.9	7,598	95.1	0.006*
	Placebo	7,946	317	4.0	7,629	96.0	
Fever^※1^	LG2055	7,988	142	1.8	7,846	98.2	< 0.001*
	Placebo	7,946	252	3.2	7,694	96.8	

*Significant difference was observed between two groups (*p* < 0.05).

^※1^The data for 7 days after COVID-19 vaccination were excluded from the results.

### Salivary secretory IgA

**FIGURE 3:**
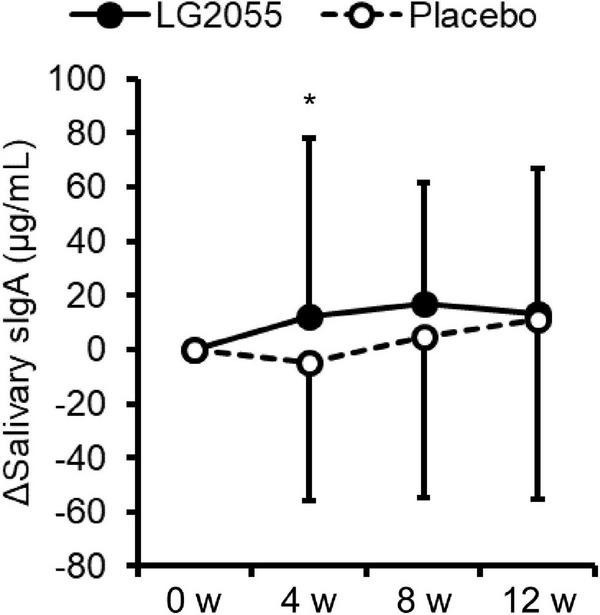
Comparison of the amount of change from baseline of salivary sIgA concentration (Δsalivary sIgA). Data are shown as mean ± SD. *p < 0.05 according to the unpaired *t*-test (LG2055 group *n* = 96 or 97; placebo group *n* = 96).

Compared with the placebo group, the LG2055 group showed significantly higher levels of changes in salivary sIgA concentration from the baseline to 4 weeks (Figure 3). However, no significant difference was detected in the measured values between the groups. In the within-group comparisons, the measured values of the LG2055 group at 8 and 12 weeks showed a significant increase from 0 week. The placebo group at 12 weeks also showed a significant increase from 0 week (Table 4).

**TABLE 4:** Measured values of the concentration of salivary sIgA during the intake period.

Parameter	Week	Group	*n*	Measured value				
				Mean	±	SD	*p* value	
							Within the group	Between the group
Salivary sIgA (μg/ml)	0	LG2055	97	130.2	±	70.7	–	0.728
		Placebo	96	137.1	±	95.1	–	
	4	LG2055	96	142.5	±	93.7	0.081	0.315
		Placebo	96	132.2	±	89.3	0.329	
	8	LG2055	96	147.9	±	72.5	<0.001^#^	0.289
		Placebo	96	142.0	±	82.1	0.152	
	12	LG2055	97	143.2	±	75.8	0.012^#^	0.866
		Placebo	96	148.0	±	85.4	0.015^#^	

^#^Significant difference was observed within the group. (Wilcoxon signed-rank test with Bonferroni correction, vs 0 w, *p* < 0.017).

### Serum derivatives of reactive oxygen metabolites and biological antioxidant potential

The LG2055 group showed significantly lower levels of serum d-ROMs than the placebo group at the end of the intake period. Moreover, the BAP/d-ROMs ratio, an index of antioxidant capacity, was significantly higher in the LG2055 group than in the placebo group (Table 5).

**TABLE 5:** Measured values of oxidative markers during the intake period.

Parameter	Week	Group	*n*	Measured value			
				Mean	±	SD	*p* value
Serum d-ROMs (U.CARR)	0	LG2055	97	299.0	±	62.7	0.085
		Placebo	96	314.8	±	72.4	
	12	LG2055	97	308.3	±	67.3	0.023*
		Placebo	96	328.9	±	71.6	
Serum BAP (μM)	0	LG2055	97	2,123.7	±	208.6	0.520
		Placebo	96	2,108.4	±	214.1	
	12	LG2055	97	2,163.9	±	153.2	0.820
		Placebo	96	2,159.1	±	139.2	
BAP/d-ROMs (μM/U.CARR)	0	LG2055	97	7.4	±	1.6	0.050
		Placebo	96	7.0	±	1.5	
	12	LG2055	97	7.3	±	1.7	0.012*
		Placebo	96	6.8	±	1.6	

*Significant difference was observed between two groups (*p* < 0.05).

### Natural killer cell activity, serum IgA, and serum IgG

No statistically significant differences were observed in the measured values and changes in NK cell activity and the concentrations of IgA and IgG in the serum (Table 6).

**TABLE 6:** Measured values of the immune markers during the intake period.

Parameter	Week	Group	*n*	Measured value			
				Mean	±	SD	*p* value
NK cell activity (%)	0	LG2055	38	57.5	±	20.5	0.272
		Placebo	39	62.5	±	18.7	
	4	LG2055	37	57.7	±	22.4	0.803
		Placebo	39	59.3	±	20.7	
	8	LG2055	37	57.9	±	22.0	0.697
		Placebo	39	60.7	±	19.3	
	12	LG2055	38	53.2	±	22.5	0.606
		Placebo	39	55.8	±	20.7	
Serum IgA (mg/dl)	0	LG2055	97	206.6	±	74.5	0.433
		Placebo	96	216.0	±	81.2	
	4	LG2055	96	205.4	±	73.6	0.591
		Placebo	96	211.3	±	78.5	
	8	LG2055	96	199.6	±	73.9	0.458
		Placebo	96	208.8	±	79.8	
	12	LG2055	97	196.8	±	69.1	0.498
		Placebo	96	203.9	±	75.5	
Serum IgG (mg/dl)	0	LG2055	97	1,169.5	±	188.1	0.385
		Placebo	96	1,194.4	±	208.3	
	4	LG2055	96	1,166.4	±	177.7	0.523
		Placebo	96	1,184.2	±	206.2	
	8	LG2055	96	1,175.6	±	188.6	0.435
		Placebo	96	1,198.3	±	212.7	
	12	LG2055	97	1,148.3	±	180.0	0.281
		Placebo	96	1,178.2	±	203.3	

### Safety assessment

During this study, 112 adverse events from 62 participants were observed. All these adverse events were judged not to be associated with the intake of the test samples by the medical doctor. Therefore, no adverse events caused by the intake of the test samples were observed.

## Discussion

In this study, we used DY containing LG2055 as the test sample and examined whether LG2055 could maintain the subjective symptoms of physical conditions through immunomodulation in healthy adults. We found that the LG2055 group showed a significantly lower ratio of the cumulative days of several local and systemic symptoms than the placebo group. In addition, changes in salivary sIgA concentration were significantly higher and the serum d-ROMs level was significantly lower in the LG2055 group. The findings suggest that the incidence rates of the symptoms associated with common cold were lesser in the LG2055 group, and LG2055 might suppress the deterioration of physical conditions by maintaining and improving the host’s immune system.

In the early phase of a cold, inflammation occurs in the upper respiratory tract, including the throat and nose. Subsequently, viral multiplication induces an immune response from the host, and systemic symptoms (e.g., fever and feeling tired) then appear. The present findings in this study suggested that ingestion of DY containing LG2055 might suppress the early local symptoms of common cold and its subsequent systemic symptoms.

It is well known that the viruses that cause common cold include influenza virus, adenovirus, coronavirus, rhinovirus, respiratory syncytial virus (RSV), and parainfluenza virus (31). The host immune system, including innate immunity and acquired immunity, plays an important role in preventing infection of these viruses. When the immune system does not work optimally, infection is easily established, and the local and systemic symptoms, such as sneezing, plugged nose, headache, and fever, appear. Especially, physical fatigue and/or various stresses suppress sIgA production and NK cell activity (32, 33), which suppress the immune system. Therefore, maintaining the immune system is expected to help prevent viral infection and suppress common cold symptoms.

LAB have been estimated to be useful for maintaining the immune system, and several studies have been performed to assess this. For example, intake of *Lactobacillus pentosus* (recently reclassified as *Lactiplantibacillus pentosus*) strain b240 has been shown to increase salivary sIgA and decrease the incidence rate of common cold (34–37); intake of *Lactococcus lactis* susp. *lactis* JCM5805 has been shown to stimulate plasmacytoid dendritic cells-related anti-viral immunity and mitigated the incidence of common cold symptoms (29, 30); *Lactobacillus paracasei* (reclassified as *Lacticaseibacillus paracasei*). MCC1849 has been shown to improve the incidence, reduce the total number of days of the symptoms, and decrease the symptom scores of common cold (37, 38). In general, it is assumed that LAB show immunostimulating effects via activation of intestinal immunity, although its detailed mechanisms are still unclear. According to one hypothesis, orally administrated LAB are captured by M cells in Peyer’s patches of the intestine and recognized by dendritic cells and/or macrophages. Subsequently, these cells are activated and promote the activation of innate and adaptive immune responses via cytokine production (39). Eventually, activated immune cells migrate to the whole body through lymphatic and circulatory systems to contribute to the protection against viral infection. Therefore, recognition of LAB by immune cells is thought to be necessary for the immunostimulating effects of LAB.

The preventive effects of LG2055 against several viruses have been reported. For example, Nakayama et al. observed the upregulated expression of anti-viral genes, the increased survival rate, and the decreased influenza A virus titer in the lungs of LG2055-administered mice (24). Administration of LG2055 also suppressed the loss of body weight caused by RSV infection and decreased the RSV titer in the lung (25). Moreover, LG2055 induced the upregulation of intestinal sIgA production in mice (23). In addition, our previous clinical study showed that changes in salivary sIgA and NK cell activity from baseline and expression of anti-viral gene (myxovirus resistance protein A) were significantly higher in the LG2055 group than in the placebo group (16).

The findings mentioned above suggest that LG2055 might improve the immune system of the host, reduce the risk of infection from common cold viruses, and/or suppress the symptoms via increased sIgA production, NK cell activity, and expression of anti-viral genes.

Actually, changes in the sIgA level were higher in the LG2055 group in this study. sIgA, which exists in the body surface and various secretory fluids, prevents the invasion of bacteria and viruses into the body (40). sIgA is an important defense system of the mucosal surface, which is called “mucosal immunity.” It is known that sIgA has the ability to bind to various viruses and bacteria and thus is effective for preventing the entry of various pathogens. Notably, studies have observed a reduction of salivary sIgA levels before upper respiratory infections, indicating that maintaining salivary sIgA levels is important to prevent infection from common cold viruses (32). These data suggest that LG2055 upregulates salivary sIgA production and contributes to protection from viral infection. Indeed, our previous study suggests that LG2055 upregulates IgA production via recognition of LG2055 by dendritic cells (23), and the increased interferon production in macrophages is important for preventing influenza A virus infection by LG2055 (24). Taken together, the recognition of LG2055, as well as other strains of LAB, by dendritic cells and/or macrophages might play important roles in the maintenance and improvement of host immune systems. Although the difference in the sIgA levels between the two groups became non-significant at 12 weeks after starting intake, it could have been influenced by the large change in the sIgA level in the placebo group.

On the other hand, a significant difference was not observed in NK cell activity between the LG2055 and placebo groups in this study. In a previous study, changes in NK cell activity from baseline were significantly higher in the LG2055 group than in the placebo group 7 weeks after the start of intake, but not at 16 weeks after the start of intake (16). Notably, there is a difference in the time points for the measurements of NK cell activities between these two clinical studies. In the present study, intake of LG2055 was performed from January 2022 to April 2022, and NK cell activities were measured at 0, 4, 8, and 12 weeks after starting intake. Whereas, the previous study was conducted from November 2014 to March 2015, and the measurements were performed at 0, 7, and 16 weeks after starting intake. It has been demonstrated that NK cell activity shows a seasonal variation (41). Thus, the difference in the time points might have affected the results. Additional investigations are needed to confirm our hypothesis.

Moreover, oxidative stress supposedly plays an important role in common cold virus infections. Actually, it is known that some viruses increase oxidative stress with their infection via upregulation of reactive oxygen species (ROS) production (42, 43). Accordingly, several studies have attempted to suppress the infection or the symptoms caused by the viruses by decreasing oxidative stress. For example, N-acetylcysteine and glutathione, the representative antioxidants, were reported to prevent the infection of influenza viruses *in vitro* and *in vivo* (44, 45). Furthermore, it was reported that both ascorbic acid and zinc were helpful in ameliorating common cold symptoms (46, 47). However, the detailed relationship between oxidative stress and imbalance of antioxidant defense of the host under viral infection remains unclear; although, it is certain that oxidative stress contributes to the viral infection.

We have previously reported the anti-oxidative effects of LG2055. In *Caenorhabditis elegans* (*C. elegans*), the resistance against oxidative stress was strengthened, and the lifespan was significantly prolonged by feeding LG2055. The findings suggest that the enhanced resistance against oxidative stress contributes to the extended lifespan of *C. elegans* (21). Furthermore, decreased ROS accumulation and increased expression of antioxidant enzymes were observed in LG2055-treated murine cells, suggesting the possibility that LG2055 would be effective in strengthening the defense system against oxidative stress not only in *C. elegans* but also in mammals (22).

In this study, the serum d-ROMs level in the LG2055 group was significantly lower than that in the placebo group and the BAP/d-ROMs ratio was also higher in the LG2055 group at 12 weeks after starting intake. Serum d-ROMs and BAP are useful oxidative stress markers. Notably, the d-ROMs test does not directly measure ROS and/or free radicals, which are the cause of oxidative stress; however, it determines the hydroperoxide metabolite level (48). Serum BAP can be used to qualify the antioxidant capacity of the blood (49). Therefore, a lower d-ROMs level indicates lower oxidative stress, and a higher BAP level indicates a higher concentration of antioxidants. In addition, the BAP/d-ROMs ratio is used as the index of antioxidant capacity (50–52). Our results suggest that intake of LG2055 strengthened the antioxidant capacity and suppressed oxidative stress.

On the other hand, as described above, viral infection increases oxidative stress; thus, it is possible that the low d-ROMs level might be due to an improved host immune system by LG2055 that was attempting to prevent viral infection. It is difficult to determine whether the observed lower d-ROMs level is the cause or consequence of the prevention of viral infection. Further studies are needed to evaluate the relationship between oxidative stress and infection by time-course measurements to reveal the underlying mechanisms.

During this study, SARS-CoV-2 infection was rapidly spreading in Japan, and the booster (third) vaccination against SARS-CoV-2 was promoted by the Japanese government. At the time of planning this study, the third vaccination had not been introduced in Japan; therefore, we set the following exclusion criteria: “The participants who did not plan to receive the COVID-19 vaccine during the ingestion period.” However, since then, the situation had changed, and we needed to prioritize ethical considerations. Thus, we allowed the participants to get inoculated with the SARS-CoV-2 vaccination during the study period and included them in the final analysis. As a result, 43 participants (24 participants in the LG2055 group and 19 participants in the placebo group) got the third vaccination during the pre-observation and intake periods. Notably, some people experience side effects, such as pain in the arm, fever, headache, muscle pain, chill, and tiredness, after the COVID-19 vaccination, and these symptoms resemble the symptoms of common cold. To eliminate the influence of side effects on the questionnaire, we excluded several symptoms (headache, feeling tired, chill, and fever) from the questionnaire results for 7 days after the vaccination. We also evaluated the questionnaire results in the case of including the data after COVID-19 vaccination or excluding the participants who got COVID-19 vaccination (Supplementary Tables 1–4). As a result, the effects of LG2055 on the subjective symptoms were observed in common, thus it was estimated that the vaccination had little influence on the results of questionnaire in this study. Moreover, we evaluated the influence of vaccination on immunological parameters, especially total IgG, which was thought to be easily affected by vaccination. Among the participants who got the vaccination during this study, serum IgG levels before and after the vaccination were compared, and no significant difference was observed (data not shown). Based on these findings, we concluded that the vaccination had an insignificant effect on the results of this study, although we needed to handle these data with great care.

In addition, the lifestyles of Japanese people, including the participants in this study, during this study were different from those in previous years because of the COVID-19 pandemic-related restrictions, such as mandatory use of masks and staying at home, and various other infection control measures. The reported number of cases of seasonal influenza in Japan significantly decreased during the 2019/2020 flu season compared with that before the COVID-19 spread (53), and a similar trend was seen in the 2021/2022 flu season; therefore, it is obvious that the Japanese people have strengthened their infection control. Further studies are needed to investigate the effects of LG2055 in a situation similar to that before COVID-19.

In conclusion, this study found that the daily intake of DY containing LG2055 for 12 weeks decreased the cumulative days of common cold symptoms in healthy adults. Moreover, changes in the salivary sIgA level were significantly higher at 4 weeks after starting intake, and the serum d-ROMs level was significantly lower at 12 weeks after starting intake in the LG2055 group. These results suggest that intake of LG2055 maintains the subjective symptoms of physical conditions by improving the host immune system.

## Data availability statement

The raw data supporting the conclusions of this article will be made available by the authors, without undue reservation.

## Ethics statement

The studies involving human participants were reviewed and approved by Ethics Committee of Nihonbashi Cardiology Clinic. The patients/participants provided their written informed consent to participate in this study.

## Author contributions

EK and TK designed the study and wrote the initial draft of the manuscript. YI was responsible for data acquisition. EK, TA, and MK conducted the study. EK, NS, and TK analyzed the data. All authors read and approved the final manuscript.
