# Supplementary material for: Intake of Lactobacillus paragasseri SBT2055 improves subjective symptoms of common cold during winter season in healthy adults: A randomized, double-blind, placebo-controlled parallel-group comparative study
Source: Front Nutr. 2022 Dec 8;9:1063584. doi: 10.3389/fnut.2022.1063584 (PMC9773393; doi:10.3389/fnut.2022.1063584)
Supplement: Supplementary file 1 [file Table_1.docx]

Supplementary Material

# Supplementary Figures and Tables

## Supplementary Figures


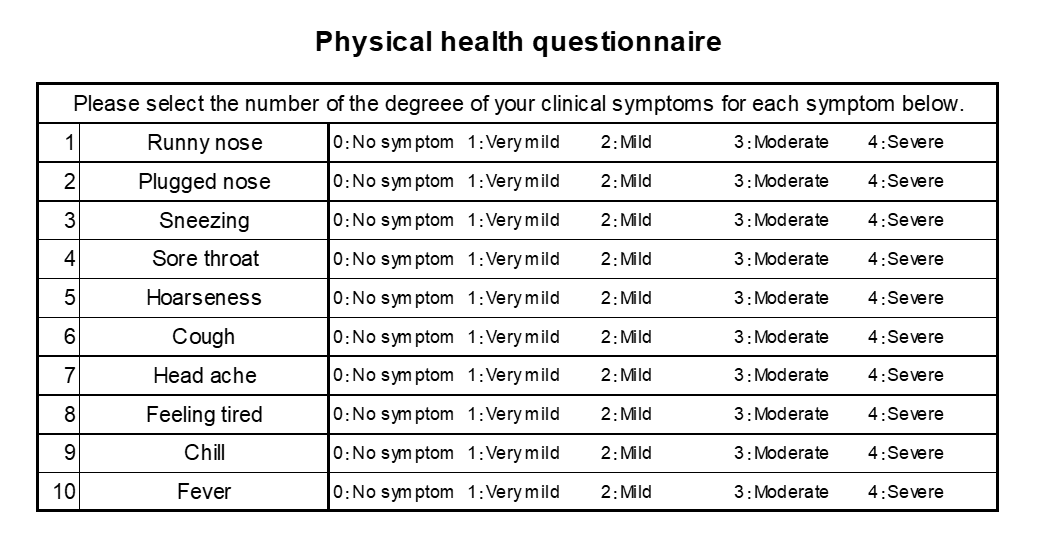


**Supplementary Figure 1.** The physical health questionnaire. The survey was conducted in Japanese.

## Supplementary Tables

**Supplementary Table 1.** Comparison of the cumulative days of each symptom (five grade) (Including the data after COVID-19 vaccination)

| Symptoms | Group | n | No  symptom | Very  mild | Mild | Moderate | Severe | *p* value |
| --- | --- | --- | --- | --- | --- | --- | --- | --- |
|  |  |  | Cumulative days  (Ratio) | | | | |  |
| Runny  nose | LG2055 | 8148 | 5764  (70.7 %) | 1826  (22.4%) | 425  (5.2%) | 110  (1.4%) | 23  (0.3%) | < 0.001^*^ |
|  | Placebo | 8066 | 5297  (65.7%) | 2013  (25.0%) | 598  (7.4%) | 148  (1.8%) | 10  (0.1%) |  |
| Plugged  nose | LG2055 | 8148 | 6702  (82.3%) | 1100  (13.5%) | 224  (2.7%) | 115  (1.4%) | 7  (0.1%) | < 0.001^*^ |
|  | Placebo | 8066 | 5830  (72.3%) | 1658  (20.6%) | 433  (5.4%) | 120  (1.5%) | 25  (0.3%) |  |
| Sneezing | LG2055 | 8148 | 6704  (82.3%) | 1046  (12.8%) | 268  (3.3%) | 118  (1.4%) | 12  (0.1%) | < 0.001^*^ |
|  | Placebo | 8066 | 6438  (79.8%) | 1252  (15.5%) | 305  (3.8%) | 66  (0.8%) | 5  (0.1%) |  |
| Sore  throat | LG2055 | 8148 | 7633  (93.7%) | 385  (4.7%) | 80  (1.0%) | 32  (0.4%) | 18  (0.2%) | < 0.001^*^ |
|  | Placebo | 8066 | 7078  (87.8%) | 754  (9.3%) | 186  (2.3%) | 48  (0.6%) | 0  (0.0%) |  |
| Hoarseness | LG2055 | 8148 | 7859  (96.5%) | 236  (2.9%) | 33  (0.4%) | 9  (0.1%) | 11  (0.1%) | < 0.001^*^ |
|  | Placebo | 8066 | 7402  (91.8%) | 480  (6.0%) | 140  (1.7%) | 41  (0.5%) | 3  (0.0%) |  |
| Cough | LG2055 | 8148 | 7709  (94.6%) | 331  (4.1%) | 71  (0.9%) | 35  (0.4%) | 2  (0.0%) | < 0.001^*^ |
|  | Placebo | 8066 | 7393  (91.7%) | 508  (6.3%) | 145  (1.8%) | 16  (0.2%) | 4  (0.0%) |  |
| Headache | LG2055 | 8148 | 7323  (89.9%) | 499  (6.1%) | 190  (2.3%) | 106  (1.3%) | 30  (0.4%) | < 0.001^*^ |
|  | Placebo | 8066 | 6968  (86.4%) | 740  (9.2%) | 261  (3.2%) | 80  (1.0%) | 17  (0.2%) |  |
| Feeling  tired | LG2055 | 8148 | 7187  (88.2%) | 667  (8.2%) | 196  (2.4%) | 75  (0.9%) | 23  (0.3%) | < 0.001^*^ |
|  | Placebo | 8066 | 6534  (81.0%) | 1087  (13.5%) | 248  (3.1%) | 147  (1.8%) | 50  (0.6%) |  |
| Chill | LG2055 | 8148 | 7751  (95.1%) | 290  (3.6%) | 81  (1.0%) | 22  (0.3%) | 4  (0.0%) | 0.218 |
|  | Placebo | 8066 | 7737  (95.9%) | 261  (3.2%) | 54  (0.7%) | 14  (0.2%) | 0  (0.0%) |  |
| Fever | LG2055 | 8148 | 7988  (98.0%) | 113  (1.4%) | 28  (0.3%) | 15  (0.2%) | 4  (0.0%) | < 0.001^*^ |
|  | Placebo | 8066 | 7794  (96.6%) | 220  (2.7%) | 33  (0.4%) | 16  (0.2%) | 3  (0.0%) |  |

*Significant difference was observed between two groups (*p* < 0.05)

**Supplementary Table 2.** Comparison of the cumulative days of each symptom (two grade) (Including the data after COVID-19 vaccination)

| Symptoms | Group | n | With symptom | | Without symptom | | *p* value |
| --- | --- | --- | --- | --- | --- | --- | --- |
|  |  |  | Cumulative  days | Ratio | Cumulative  days | Ratio |  |
| Runny  nose | LG2055 | 8148 | 2384 | 29.3% | 5764 | 70.7% | < 0.001^*^ |
|  | Placebo | 8066 | 2769 | 34.3% | 5297 | 65.7% |  |
| Plugged  nose | LG2055 | 8148 | 1446 | 17.7% | 6702 | 82.3% | < 0.001^*^ |
|  | Placebo | 8066 | 2236 | 27.7% | 5830 | 72.3% |  |
| Sneezing | LG2055 | 8148 | 1444 | 17.7% | 6704 | 82.3% | < 0.001^*^ |
|  | Placebo | 8066 | 1628 | 20.2% | 6438 | 79.8% |  |
| Sore  throat | LG2055 | 8148 | 515 | 6.3% | 7633 | 93.7% | < 0.001^*^ |
|  | Placebo | 8066 | 988 | 12.2% | 7078 | 87.8% |  |
| Hoarseness | LG2055 | 8148 | 289 | 3.5% | 7859 | 96.5% | < 0.001^*^ |
|  | Placebo | 8066 | 664 | 8.2% | 7402 | 91.8% |  |
| Cough | LG2055 | 8148 | 439 | 5.4% | 7709 | 94.6% | < 0.001^*^ |
|  | Placebo | 8066 | 673 | 8.3% | 7393 | 91.7% |  |
| Headache | LG2055 | 8148 | 825 | 10.1% | 7323 | 90.2% | < 0.001^*^ |
|  | Placebo | 8066 | 1098 | 13.6% | 6968 | 86.7% |  |
| Feeling  tired | LG2055 | 8148 | 961 | 11.8% | 7187 | 88.2% | < 0.001^*^ |
|  | Placebo | 8066 | 1532 | 19.0% | 6534 | 81.0% |  |
| Chill | LG2055 | 8148 | 397 | 4.9% | 7751 | 95.1% | 0.015^*^ |
|  | Placebo | 8066 | 329 | 4.1% | 7737 | 95.9% |  |
| Fever | LG2055 | 8148 | 160 | 2.0% | 7988 | 98.0% | < 0.001^*^ |
|  | Placebo | 8066 | 272 | 3.4% | 7794 | 96.6% |  |

*Significant difference was observed between two groups (*p* < 0.05)

**Supplementary Table 3.** Comparison of the cumulative days of each symptom (five grade) (Excluding the participants who got COVID-19 vaccination)

| Symptoms | Group | n | No  symptom | Very  mild | Mild | Moderate | Severe | *p* value |
| --- | --- | --- | --- | --- | --- | --- | --- | --- |
|  |  |  | Cumulative days  (Ratio) | | | | |  |
| Runny  nose | LG2055 | 6132 | 4283  (69.8 %) | 1507  (24.6%) | 296  (4.8%) | 40  (0.7%) | 6  (0.1%) | < 0.001^*^ |
|  | Placebo | 6470 | 4256  (65.8%) | 1637  (25.3%) | 442  (6.8%) | 126  (1.9%) | 9  (0.1%) |  |
| Plugged  nose | LG2055 | 6132 | 5063  (82.6%) | 871  (14.2%) | 157  (2.6%) | 38  (0.6%) | 3  (0.0%) | < 0.001^*^ |
|  | Placebo | 6470 | 4550  (70.3%) | 1435  (22.2%) | 347  (5.4%) | 113  (1.7%) | 25  (0.4%) |  |
| Sneezing | LG2055 | 6132 | 5133  (83.7%) | 784  (12.8%) | 186  (3.0%) | 29  (0.5%) | 0  (0.0%) | < 0.001^*^ |
|  | Placebo | 6470 | 5227  (80.8%) | 968  (15.0%) | 213  (3.3%) | 58  (0.9%) | 4  (0.1%) |  |
| Sore  throat | LG2055 | 6132 | 5703  (93.0%) | 323  (5.3%) | 67  (1.1%) | 23  (0.4%) | 16  (0.3%) | < 0.001^*^ |
|  | Placebo | 6470 | 5659  (87.5%) | 623  (9.6%) | 142  (2.2%) | 46  (0.7%) | 0  (0.0%) |  |
| Hoarseness | LG2055 | 6132 | 5887  (96.0%) | 202  (3.3%) | 29  (0.5%) | 4  (0.1%) | 10  (0.2%) | < 0.001^*^ |
|  | Placebo | 6470 | 5928  (91.6%) | 387  (6.0%) | 113  (1.7%) | 39  (0.6%) | 3  (0.0%) |  |
| Cough | LG2055 | 6132 | 5815  (94.8%) | 230  (3.8%) | 57  (0.9%) | 28  (0.5%) | 2  (0.0%) | < 0.001^*^ |
|  | Placebo | 6470 | 5948  (91.9%) | 374  (5.8%) | 128  (2.0%) | 16  (0.2%) | 4  (0.1%) |  |
| Headache | LG2055 | 6132 | 5603  (91.4%) | 338  (5.5%) | 121  (2.0%) | 57  (0.9%) | 13  (0.2%) | < 0.001^*^ |
|  | Placebo | 6470 | 5731  (88.6%) | 470  (7.3%) | 196  (3.0%) | 58  (0.9%) | 15  (0.2%) |  |
| Feeling  tired | LG2055 | 6132 | 5319  (86.7%) | 594  (9.7%) | 164  (2.7%) | 42  (0.7%) | 13  (0.2%) | < 0.001^*^ |
|  | Placebo | 6470 | 5306  (82.0%) | 791  (12.2%) | 194  (3.0%) | 129  (2.0%) | 50  (0.8%) |  |
| Chill | LG2055 | 6132 | 5772  (94.1%) | 258  (4.2%) | 78  (1.3%) | 20  (0.3%) | 4  (0.1%) | 0.006^*^ |
|  | Placebo | 6470 | 6244  (96.5%) | 172  (2.7%) | 43  (0.7%) | 11  (0.2%) | 0  (0.0%) |  |
| Fever | LG2055 | 6132 | 5996  (97.8%) | 96  (1.6%) | 25  (0.4%) | 11  (0.2%) | 4  (0.1%) | < 0.001^*^ |
|  | Placebo | 6470 | 6229  (96.3%) | 200  (3.1%) | 27  (0.4%) | 11  (0.2%) | 3  (0.0%) |  |

*Significant difference was observed between two groups (*p* < 0.05)

**Supplementary Table 4.** Comparison of the cumulative days of each symptom (two grade) (Excluding the participants who got COVID-19 vaccination)

| Symptoms | Group | n | With symptom | | Without symptom | | *p* value |
| --- | --- | --- | --- | --- | --- | --- | --- |
|  |  |  | Cumulative  days | Ratio | Cumulative  days | Ratio |  |
| Runny  nose | LG2055 | 6132 | 1849 | 30.2% | 4283 | 69.8% | < 0.001^*^ |
|  | Placebo | 6470 | 2214 | 34.2% | 4256 | 65.8% |  |
| Plugged  nose | LG2055 | 6132 | 1069 | 17.4% | 5063 | 82.6% | < 0.001^*^ |
|  | Placebo | 6470 | 1920 | 29.7% | 4550 | 70.3% |  |
| Sneezing | LG2055 | 6132 | 999 | 16.3% | 5133 | 83.7% | < 0.001^*^ |
|  | Placebo | 6470 | 1243 | 19.2% | 5227 | 80.8% |  |
| Sore  throat | LG2055 | 6132 | 429 | 7.0% | 5703 | 93.0% | < 0.001^*^ |
|  | Placebo | 6470 | 811 | 12.5% | 5659 | 87.5% |  |
| Hoarseness | LG2055 | 6132 | 245 | 4.0% | 5887 | 96.0% | < 0.001^*^ |
|  | Placebo | 6470 | 542 | 8.4% | 5928 | 91.6% |  |
| Cough | LG2055 | 6132 | 317 | 5.2% | 5815 | 94.8% | < 0.001^*^ |
|  | Placebo | 6470 | 522 | 8.1% | 5948 | 91.9% |  |
| Headache | LG2055 | 6132 | 529 | 8.6% | 5603 | 91.4% | < 0.001^*^ |
|  | Placebo | 6470 | 739 | 11.4% | 5731 | 88.6% |  |
| Feeling  tired | LG2055 | 6132 | 813 | 13.3% | 5319 | 86.7% | < 0.001^*^ |
|  | Placebo | 6470 | 1164 | 18.0% | 5306 | 82.0% |  |
| Chill | LG2055 | 6132 | 360 | 5.9% | 5772 | 94.1% | < 0.001^*^ |
|  | Placebo | 6470 | 226 | 3.5% | 6244 | 96.5% |  |
| Fever | LG2055 | 6132 | 136 | 2.2% | 5996 | 97.8% | < 0.001^*^ |
|  | Placebo | 6470 | 241 | 3.7% | 6229 | 96.3% |  |

*Significant difference was observed between two groups (*p* < 0.05)
